# Construction of an immune-related lncRNA signature as a novel prognosis biomarker for LUAD

**DOI:** 10.18632/aging.203455

**Published:** 2021-08-26

**Authors:** Hang Chen, Wei Shen, Saiqi Ni, Menglu Sang, Shibo Wu, Yinyu Mu, Kaitai Liu, Ni Li, Linwen Zhu, Guodong Xu

**Affiliations:** 1 Medical School, Ningbo University, Ningbo, Zhejiang, China; 2 Department of Pulmonary and Critical Care Medicine, The Third People’s Hospital of Cixi, Ningbo, Zhejiang, China; 3 Department of Urology, Ningbo City First Hospital, Ningbo, Zhejiang, China; 4 Department of Cardiothoracic Surgery, The Affiliated Lihuili Hospital, Ningbo University, Ningbo, Zhejiang, China

**Keywords:** lung adenocarcinoma, long noncoding RNA, tumor immune microenvironment, prognostic signature, infiltration of immune cell

## Abstract

The tumor immune microenvironment of lung cancer is associated with prognosis and immunotherapy efficacy. Long noncoding RNAs are identified as prognostic biomarkers associated with immune functions. We constructed a signature comprising differentially expressed immune-related lncRNAs to predict the prognosis of patients with lung adenocarcinoma. We established the immune-related lncRNA signature by pairing immune-related lncRNAs regardless of expression level and lung adenocarcinoma patients were divided into high- and low-risk groups. The prognosis of patients in the two groups was significantly different; The immune-related lncRNA signature could serve as an independent lung adenocarcinoma prognostic indicator. The signature correlated negatively with B cell, CD4+ T cell, M2 macrophage, neutrophil, and monocyte immune infiltration. Patients with low risk scores had a higher abundance of immune cells and stromal cells around the tumor. Gene set enrichment analysis showed that samples from low-risk group were more active in the IgA production in intestinal immune network and the T and B cell receptor signaling pathway. High-risk groups had significant involvement of the cell cycle, DNA replication, adherens junction, actin cytoskeleton regulation, pathways in cancer, and TGF-β signaling pathways. High risk scores correlated significantly negatively with high CTLA-4 and HAVCR2 expression and higher median inhibitory concentration of common anti-tumor chemotherapeutics (e.g., cisplatin, paclitaxel, gemcitabine) and targeted therapy (e.g., erlotinib and gefitinib). We identified a reliable immune-related lncRNA lung adenocarcinoma prognosis model, and the immune-related lncRNA signature showed promising clinical prediction value.

## INTRODUCTION

According to the latest research, lung cancer is the leading malignant tumor in China and even in the world. [1–3] Lung adenocarcinoma (LUAD) is the major type of non–small cell lung cancer (NSCLC) [4]. Clinical studies have reported that the tumor immune microenvironment is involved in tumorigenesis [5] and plays vital roles in the prognosis of patients. For example, increasing evidence has demonstrated that the macrophages infiltration are decisive in the lung tumor immune microenvironment (TME), leading to poor survival [6]. Ye et al. [7] revealed that PECAM1 is involved in LUAD development by increasing the abundance of several immune cells. Han et al. [8] constructed a prognosis model comprising several B lymphocyte-specific genes to predict the prognosis and immunotherapy and radiotherapy responses in patients with LUAD.

Long noncoding RNAs (lncRNAs) are a type of non-coding RNA molecules with transcripts of >200 nucleotides [9]. They can regulate the expression of downstream genes by physically interacting with DNA, RNA, or protein. Therefore, lncRNAs had a significant impact on the occurrence, progression of cancers [10, 11]. Recently, utilizing immune-related lncRNA (irlncRNAs) to predict the prognosis of patients has become a research hotspot. Yu et al. [12] found that the lncRNA FAM207BP facilitated LUAD cell proliferation, migration, and invasion. Li et al. [4] constructed a 7-irlncRNA model to increase the predictive value for LUAD. Lv et al. [13] proposed that dual-gene markers are more sensitive than single-gene markers in the early diagnosis of malignant tumors. In addition, clinical work sometimes involves gene expression data such as that from PCR and microarrays instead of transcriptome data. Here, we used an improved modeling algorithm to construct a prognosis model based on differentially expressed immune-related lncRNAs (DEirlncRNAs) pairs without needing to accurately measure the expression levels, which avoided normalization during the conversion between data and may be indicative of the prognostic markers in LUAD patients.

## MATERIALS AND METHODS

### Data acquisition

We obtained the LUAD transcriptome profiles and the corresponding clinical information of LUAD patients and normal samples from The Cancer Genome Atlas (TCGA, https://portal.gdc.cancer.gov/) database in December 2020. We excluded samples with repeated data and those with a follow-up time of 0 days. We downloaded gene transfer format (GTF) files from Ensembl (http://asia.ensembl.org) to differentiate lncRNAs and mRNAs. We also downloaded several immune-related genes from The Immunology Database and Analysis Portal (ImmPort, https://www.immport.org/). We calculated the correlation coefficient between the immune-related genes and lncRNAs to identify irlncRNAs. The thresholds were set as |cor| > 0.4 with *p* < 0.001. To identify the differentially expressed irlncRNAs (DEirlncRNAs), we performed the R-x64-4.0.3 limma package for irlncRNA differential expression analysis (log fold change [FC] > 1, false discovery rate [FDR] < 0.05).

### Matching DEirlncRNA pairs

The DEirlncRNAs were singly cyclically matched and a 0- or -1 matrix was constructed supposing A was a DEirlncRNA pair composed of lncRNA X and lncRNA Y; A was defined as 0 if the expression level of lncRNA X was lower than that of lncRNA Y; otherwise, A was defined as 1. Next, the matrix was further filtered. Regardless of whether its expression was 0 or 1, it could not directly predict the patient’s prognosis. When the number of DEirlncRNAs pairs with 0 or 1 expression accounted for <20% of the total pairs, they were excluded.

### Construction of DEirlncRNA pairs and prognostic signature

Univariate Cox analysis was performed to screen the DEirlncRNA pairs whose expression levels were significantly related to the overall survival (OS) of the LUAD patients (*p* < 0.05). The modified least absolute shrinkage and selection operator (LASSO) regression was used to identify the DEirlncRNA pairs most relevant to OS by running the R-x64-4.0.3 glmnet package. The modified Lasso regression was run with 10-fold cross-validation (*p* < 0.05) and was set 1000 cycles with 1000 random stimulation for each cycle. Subsequently, the occurrence frequency of every pair in the Lasso regression was counted, and pairs with >100 frequency were chosen for subsequent Cox proportional hazard regression analysis. Then, the receiver operator characteristic curve (ROC) was plotted and the area under the curve (AUC) was counted. The model’s 1-, 2-, and 3-year ROC were plotted as well. The risk score with the constructed prognosis signature was calculated for every clinical sample with the formula as follows:

$$\begin{array}{c} \text{n}\\ \text{Risk score }=\;\sum \text{Coef }\left(\text{i}\right)\;\times \text{E}\left(\text{i}\right),\\ \text{i }=\text{1}, \end{array}$$

Coef (i) and E(i) represent the regression coefficient of the multivariate Cox analysis for the DEirlncRNA pairs and the value of each DEirlncRNA pair expression, respectively. The maximum inflection point of the 1-year ROC curve was identified by determining the maximum Akaike information criterion (AIC) values, and it was recognized as the cut-off point for dividing each sample into different risk groups. The value of Youden index (Sensitivity+Specificity−1) is the largest at the maximum inflection point.

### Validation of the risk prognosis model

Kaplan–Meier log-rank test was performed for comparing the survival between different risk groups to evaluate the predictive value of the prognostic signature. The specific risk scores of every patient were calculated by R-x64-4.0.3 packages survival, glmnet, survivalROC, and survminer. Several chi-square tests were performed to explore the correlation between the risk score and the clinicopathological characteristics. The figures was labeled as follows for visualization: ***<0.001, **<0.01, and *<0.05. The risk score differences in clinicopathological characteristics among different groups were calculated performing the Wilcoxon signed-rank test. Univariate and multivariate Cox regression analyses were used to validate whether the constructed model was an independent prognostic indicator for LUAD patients. The results were demonstrated with a forest map. The R-x64-4.0.3 packages utilized here were survival, pHeatmap, and ggupbr.

### Correlation analysis of tumor immune microenvironment

Considering that the six DEirlncRNA pairs were associated with tumor immunity, we calculated the correlation coefficient between the risk prognosis model and the LUAD-infiltrating immune cells based on TIMER, xCELL, quanTIseq, MCPcounter, EPIC, CIBERSORT−ABS, and CIBERSORT software. The correlation coefficients are shown in a lollipop diagram. The differences in tumor-infiltrating immune cell abundance between different risk groups were explored by Wilcoxon signed-rank test. Spearman correlation analysis was used to calculate the correlation coefficients between the risk scores and the tumor-infiltrating immune cells using the R-x64-4.0.3 ggplot2 package. To further explore the TME of patients with LUAD, we calculated the patients’ StromalScore, ImmuneScore, and ESTIMATEScore (StromalScore + ImmuneScore) using the R-x64-4.0.3 limma and estimate packages. Next, the differences in the stromal cell and immune cell content around the tumor between different risk groups were calculated by the Wilcoxon signed-rank tests. Column diagrams were drawn with GraphPad Prism 8.0.2 for visualization.

### Gene set enrichment analysis (GSEA)

We used GSEA 4.0.1 (https://www.gsea-msigdb.org/gsea/index.jsp) [14] to screen functional pathways in different risk groups. The two risk groups were performed on Kyoto Encyclopedia of Genes and Genomes (KEGG) gene sets. The thresholds were set as *p* < 0.05 and FDR < 0.25, a random sample permutation was set for 1000. All other settings were set according to their default values.

### Expression analysis of immune checkpoint inhibitor (ICIs)-related immunosuppressive molecules

We used the R-x64-4.0.3 packages ggpubr and limma to analyze the differences in ICI-related immunosuppressive molecules’ expression level between different risk groups of the constructed model, and visualized the result with a violin plot.

### The significance of the signature for guiding medication

National Comprehensive Cancer Network guidelines recommend anti-tumor drugs such as cisplatin, paclitaxel, gemcitabine, gefitinib, and erlotinib for treating lung cancer. To better guide our clinical medication, we calculated the median inhibitory concentration (IC_50_) of common anti-tumor drugs used for treating LUAD. Wilcoxon signed-rank test was performed to analyze the difference in the IC_50_ between different risk groups. We used the R-x64-4.0.3 pRRophetic and ggplot2 packages and visualized the results with several box drawings.

### Consent for publication

Written informed consent for publication was obtained from all participants.

### Availability of data and materials

All data analysed during the current study are accessible from the TCGA database (https://portal.gdc.cancer.gov/).

## RESULTS

### Identification of DEirlncRNAs

**Figure 1:**
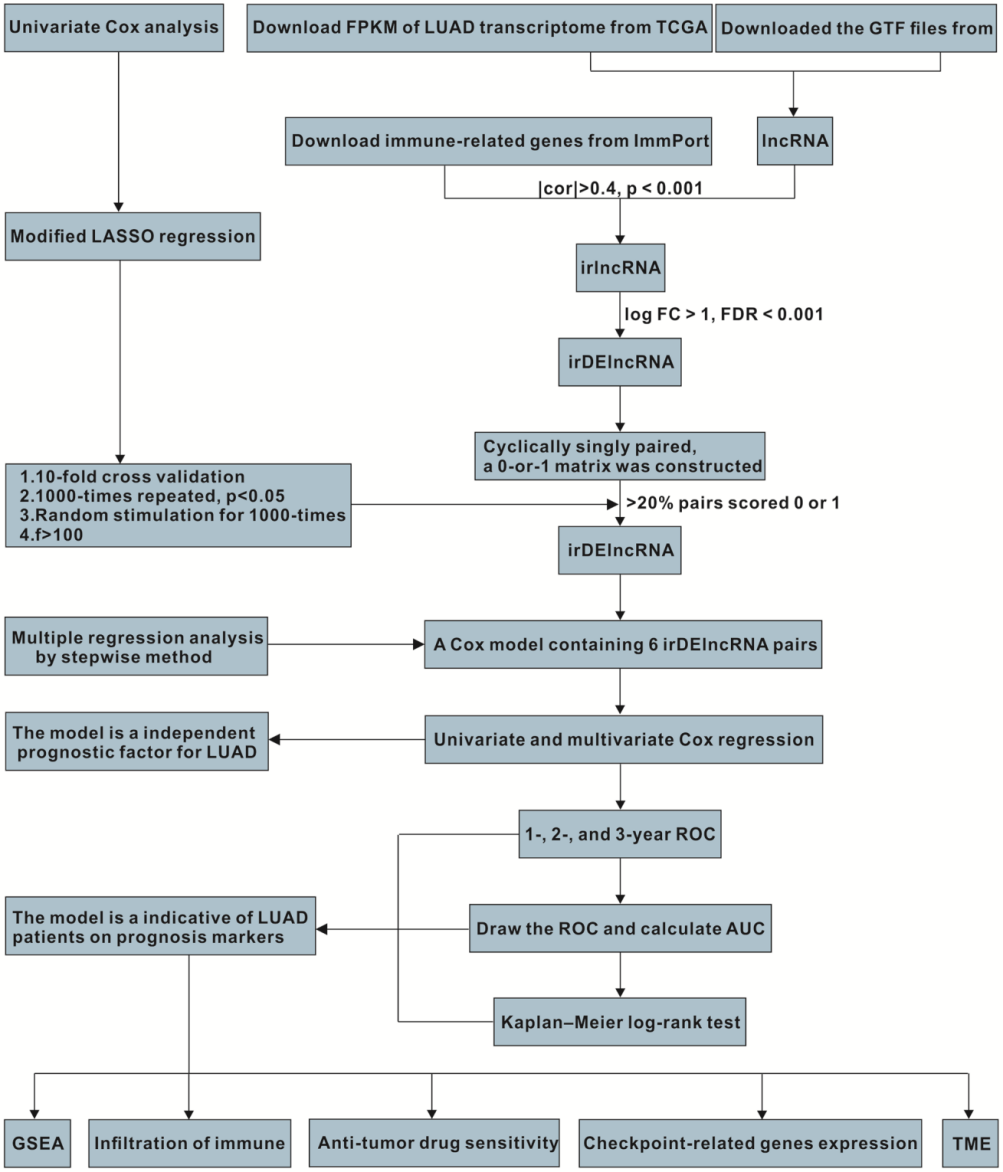
The work flowchart for the study.

**Figure 2:**
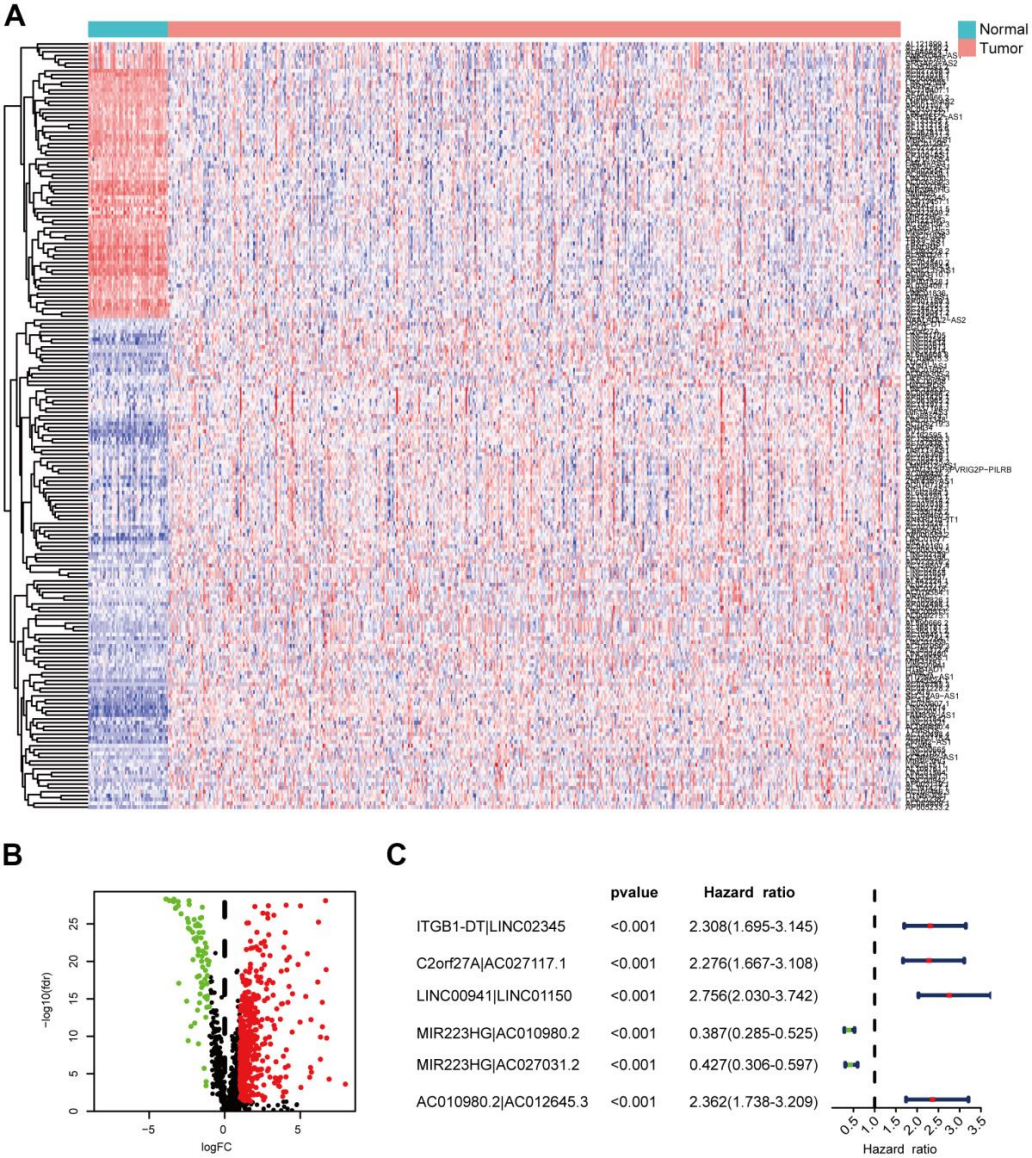
**Identification of DEirlncRNAs.** (**A**, **B**) The differential co-expression analysis identified 517 DEirlncRNAs. (**C**) Forest map shows the six DEirlncRNA pairs screened by Cox proportional hazard regression.

We identified DEirlncRNAs using a multi-step approach (Figure 1). We downloaded LUAD transcriptome data and corresponding clinical data from TCGA-LUAD cohort (54 normal samples and 497 LUAD samples). Then, we annotated the mRNAs and lncRNAs using Ensembl GTF files, and downloaded the immune-related genes. Next, we identified the irlncRNAs from the list of immune-related genes using co-expression analysis. Finally, 517 DEirlncRNAs were identified using R-x64-4.0.3 language (Figure 2A): 72 were downregulated and 445 were upregulated (Figure 2B).

### Construction of the prognostic signature

**Figure 3:**
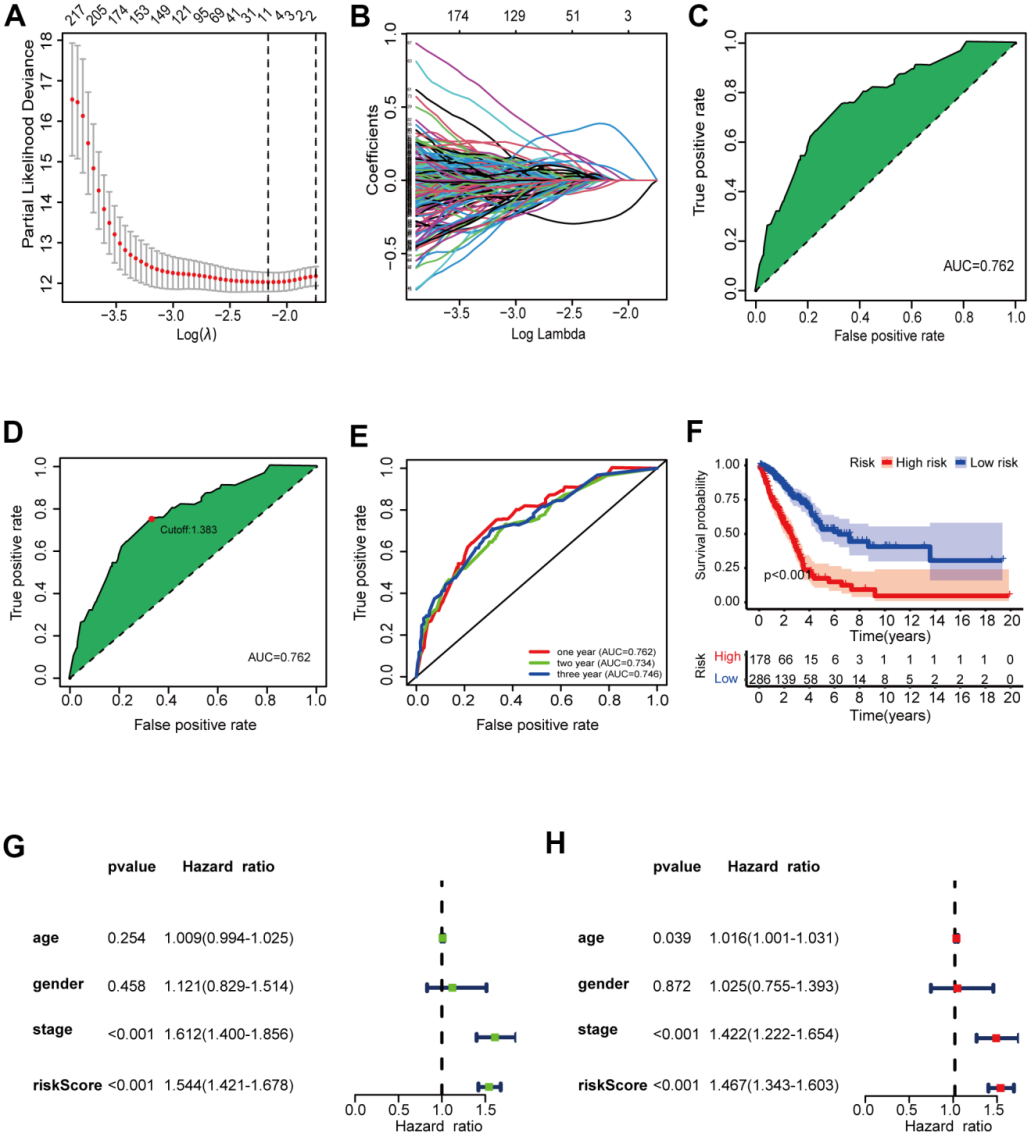
**Validation of the 6-DEirlncRNA pair signature.** (**A**, **B**) The modified LASSO penalized regression analysis identified eight DEirlncRNA pairs most related to prognostics. (**C**, **D**) The ROC showed the optimal cut-off point based on AIC value. (**E**) The 1-, 2-, 3-year AUC values were >0.7. (**F**) High-risk patients exhibited worse OS than the low-risk patients. (**G**, **H**) Univariate (**G**) and multivariate (**H**) Cox regression analysis of risk score, age, sex, and tumor-node-metastasis (TNM) stage indicating that risk score (HR = 1.467 [1.343–1.603], *p* < 0.001) and stage (HR = 1.422, [CI] = [1.222–1.654], *p* < 0.001) were related to survival.

**Figure 4:**
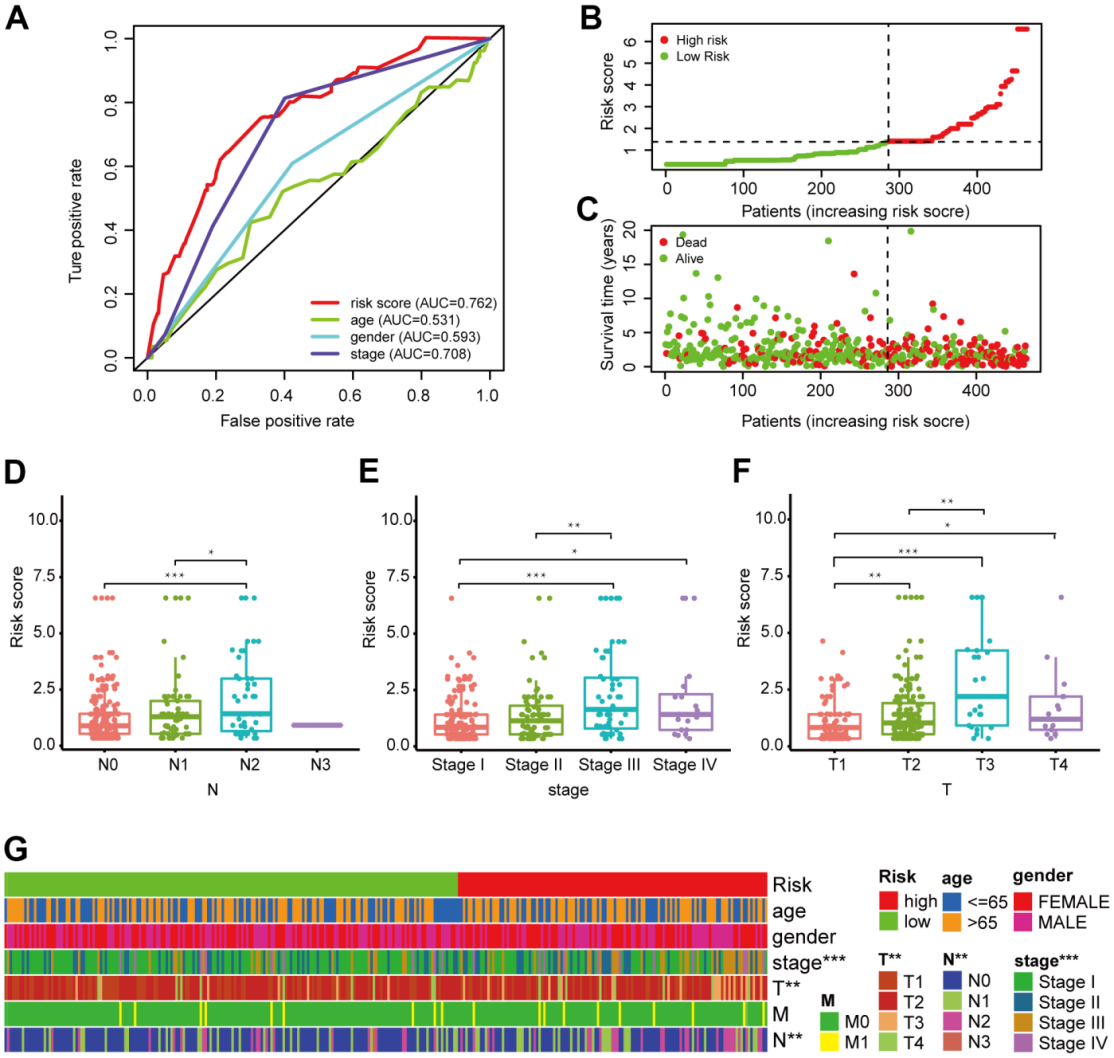
**Validation of the 6-DEirlncRNA pair signature.** (**A**) Calculation of the AUC for risk score, age, sex, and TNM stage shows that risk score is the maximum. (**B**, **C**) The risk curve (**B**) and scatter plot (**C**) indicates that patients with high risk score had worse survival. (**D**–**G**) Scatter diagrams and strip chart (**G**) show that N stage (**D**), clinical stage (**E**), and T stage (**F**) were significantly related to the risk score.

Using an iteration loop and a 0-or-1 matrix screening among 517 DEirlncRNAs, we identified 93,144 valid DEirlncRNA pairs. Univariate Cox analysis was first performed (Figure 2C). Subsequently, modified Lasso regression analysis (Figure 3A, 3B) yielded eight DEirlncRNA pairs; six were included for constructing the risk assessment model. To assess the model’s accuracy, we plotted an ROC for validation (Figure 3C). The curve indicated that the AUC for this model was 0.762. The 1-, 2-, and 3-year AUC values were 0.762, 0.734, and 0.746, respectively (Figure 3E). Compared with other clinical characteristics such as age, sex, and stage, the 1-year AUC value was the maximum (Figure 4A). According to the AIC values, the maximum inflection point on the 1-year ROC curve was recognized as the cut-off point (Figure 3D). Based on the cut-off point, the LUAD patients were distinguished into different risk groups.

### The prognostic signature is a powerful LUAD prognostic indicator

Based on the above division, there were 286 low-risk patients and 178 high-risk patients. Survival analysis (Figure 3F) indicated that the patients with high scores had a worse prognosis than patients in low-risk group (*p* < 0.001). We investigated the relationship between risk scores and clinicopathological characteristics with chi-square tests and generated a heat map. To assess the meaning of the prognosis model in clinical application, we compared the constructed model with clinicopathological characteristics using univariate (Figure 3G) and multivariate (Figure 3H) Cox analyses and ROC analysis. The results suggested that two factors, i.e., the risk score (hazard ratio [HR] = 1.467, confidence interval [CI] = 1.343–1.603, *p* < 0.001) and stage (HR = 1.422, [CI] = 1.222–1.654, *p* < 0.001), correlated with the survival. This model could also predict the survival status (AUC = 0.762; 1-year AUC = 0.762; 2-year AUC = 0.734; 3-year AUC = 0.746). The above results show that the prognostic signature can serve as a potential independent prognostic predictor.

We generated a risk curve (Figure 4B) and a scatter plot (Figure 4C) to visualize the survival status and risk score of each patient with LUAD, which indicated that patients in the high-risk group had higher risk coefficient and mortality compared to low-risk patients. Then, we investigated the correlation between the constructed model and clinicopathological characteristics using chi-square testing. The scatter diagrams and consequent strip chart (Figure 4G) indicated that N stage (Figure 4D), clinical stage (Figure 4E), and T stage (Figure 4F) were associated with the risk score significantly.

### Correlation between LUAD prognostic signature and the tumor immune microenvironment

**Figure 5:**
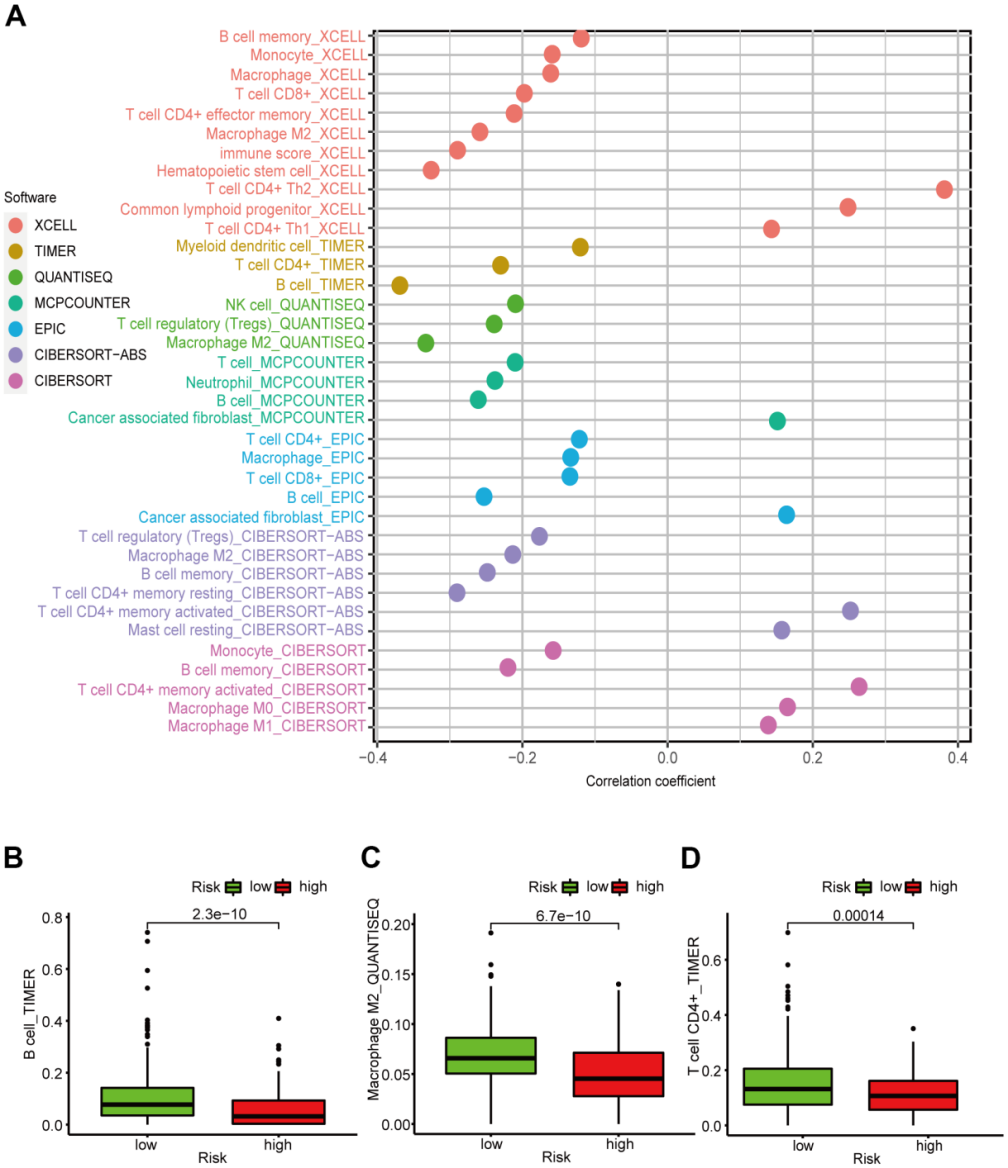
**Correlation between the LUAD 6-irlncRNA pair signature and immune cell subtype infiltration.** (**A**) Estimation of the infiltrating immune cells by TIMER, CIBERSORT, xCELL, quanTIseq, MCPcounter, EPIC, and CIBERSORT-ABS. (**B**–**D**) High-risk patients exhibited higher content of B cells (**B**), CD4+ T cells (**C**), and M2 macrophages (**D**) compared to the low-risk patients.

**Figure 6:**
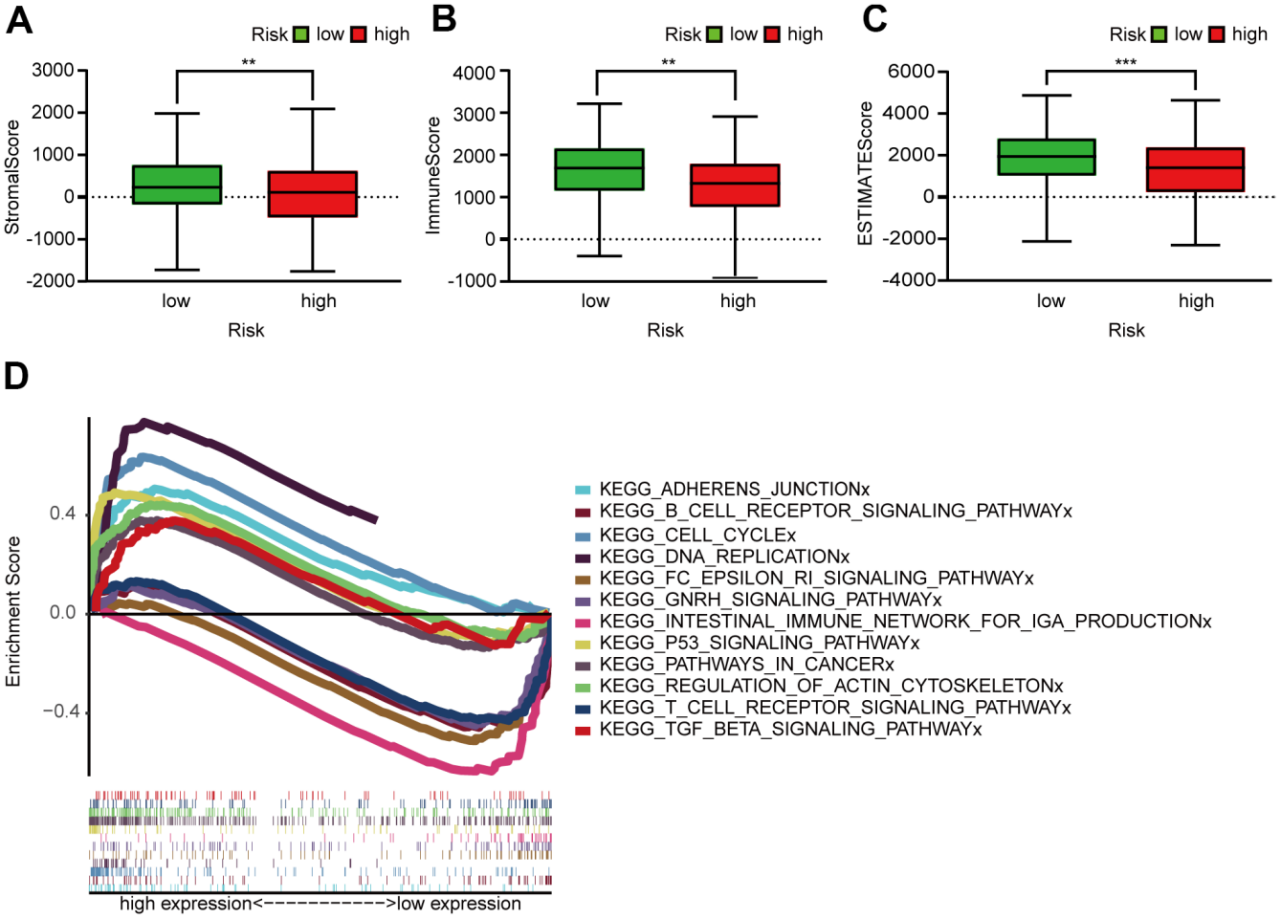
**Assessment of TME and GSEA of high- and low-risk patients.** (**A**–**C**) Column diagrams show that the high-risk group had significantly lower StromalScore (**A**), ImmuneScore (**B**), and ESTIMATEScore (**C**) than the low-risk group. (**D**) GSEA results reveal that intestinal immune network for IgA production and the T and B cell receptor signaling pathway were enriched in the low-risk groups. In the high-risk groups, cell cycle, P53 signaling, DNA replication, adherens junction, actin cytoskeleton regulation, pathways in cancer, and TGF-β signaling pathways were active relatively.

Figure 5A showed that the B cell, CD4+ T cell, M2 macrophage, neutrophil, and mast cell correlation values with the risk score were -0.368, -0.230, -0.333, -0.238, and -0.282, respectively, indicating that the abundance of these tumor-infiltrating immune cells correlated negatively with the LUAD risk score (Figure 5B–5D). We were also able to conclude that the StromalScore (Figure 6A), ImmuneScore (Figure 6B), and ESTIMATEScore (Figure 6C) associated negatively with the LUAD risk score and are all statistically significant.

### Enrichment of the functional phenotypes of the irlncRNA signature

GSEA of the prognosis model (Figure 6D) revealed that several pathways were enriched in low-risk patients: the IgA production in intestinal immune network and the T and B cell receptor signaling pathway. However, cell cycle, P53 signaling, DNA replication, adherens junction, actin cytoskeleton regulation, pathways in cancer, and TGF signaling were enriched in high-risk patients.

### The risk assessment model is a potential predictor for guiding clinical medication

**Figure 7:**
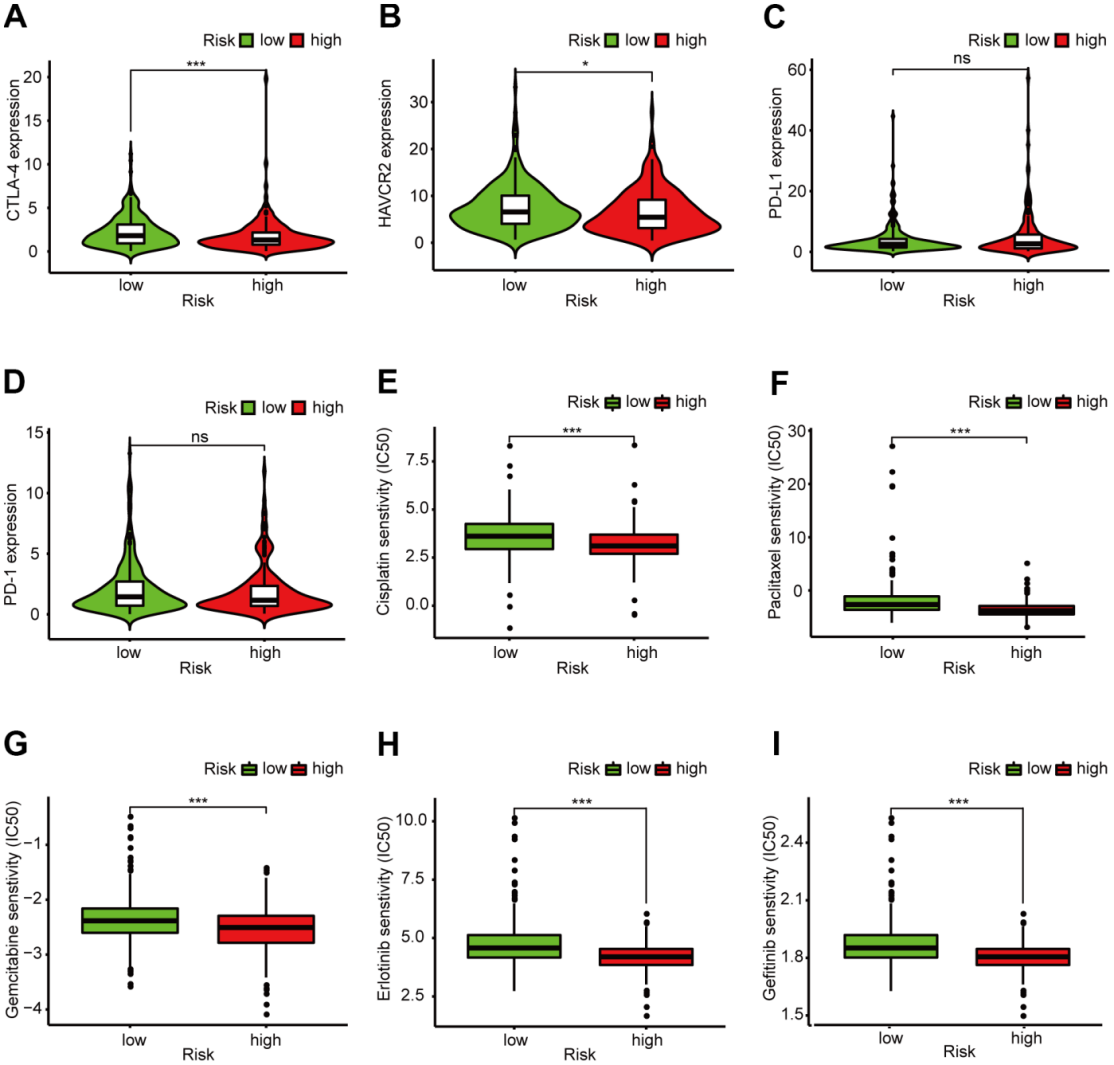
**Clinical application of the 6-DEirlncRNA pair signature.** (**A**–**D**) High risk score correlated negatively with high HAVCR2 (**B**) and PD-1 (**D**) expression, and correlated positively with high CTLA-4 (**A**) and PD-L1 (**C**) expression; There was no statistical difference in PD-1 and PD-L1 between different risk groups. (**E**–**I**) The signature could serve as a promising biomarker of anti-tumor drug sensitivity: low risk scores were associated with higher IC_50_ for cisplatin (**E**), paclitaxel (**F**), gemcitabine (**G**), erlotinib (**H**), and gefitinib (**I**).

In recent years, ICIs have been used in LUAD treatment. We determined whether there were statistical differences in ICI-related biomarkers expression between different risk groups, and discovered that a high risk score correlated negatively with high CTLA-4 (*p* < 0.001, Figure 7A), HAVCR2 (*p* < 0.05, Figure 7B), and PD-1 (*p* > 0.05, Figure 7D) expression, and correlated positively with high PD-L1 expression (*p* > 0.05, Figure 7C); There was no statistical difference in PD-1 and PD-L1 between the high- and low-risk groups. We also calculated the IC_50_ of common anti-tumor drugs in different risk groups and discovered that patients with low risk scores were correlated with a higher IC_50_ of chemotherapeutics such as cisplatin (*p* < 0.001, Figure 7E), paclitaxel (*p* < 0.001, Figure 7F), and gemcitabine (*p* < 0.001, Figure 7G) and targeted therapy such as erlotinib (*p* < 0.001, Figure 7H) and gefitinib (*p* < 0.001, Figure 7I), indicating that the prognostic signature acted as a promising predictor of anti-tumor drug sensitivity.

## DISCUSSION

Current studies utilized coding genes and ncRNAs to build predictive prognostic models for patients with lung cancer. Most prognostic models are constructed based on transcriptome expression levels [15, 16]. However, in the present study, we only needed to know which irlncRNA expression level was higher in the DEirlncRNA pair, rather than the specific expression level of each DEirlncRNA, which rendered this model applicable to all forms of gene expression levels. At present, prognostic models based on irlncRNA pairs for LUAD are still lacking.

First, we downloaded lncRNA transcriptome profiles from TCGA database, utilized differential co-expression analysis to identify DEirlncRNAs, and matched DEirlncRNA pairs performing a modified cyclical single pairing method and a 0-or-1 matrix. Next, we used univariate Cox analysis integrated with an improved Lasso penalized regression that induced cross-validation, multi-iterations, and random stimulation procedures to identify the DEirlncRNA pairs. Then, we analyzed 1-, 2-, and 3-year AUC value to obtain an ideal signature and identified the optimal cut-off point based on AIC value to differentiate the LUAD samples into different risk groups. Subsequently, we evaluated the prognostic signature in various clinical parameters, including survival, clinicopathological characteristics, infiltration of immune cells, TME, and IC_50_ of common anti-tumor drugs.

Most previous research were devoted to single lncRNA expression [17], and it is difficult to explain the complex molecular mechanisms of cancer. However, recent research have reported that multiple irlncRNAs can be combined to improve the predictive value for OS of LUAD patients. For example, Miao et al. [18] identified six irlncRNAs that could serve as an independent LUAD prognostic indicator. Wang et al. [19] demonstrated that the DEirlncRNA LINC00941 in the process of modeling leads to decreased survival in patients with LUAD. Other DEirlncRNAs, e.g., ITGB1-DT, LINC02345, C2orf27A, AC027117.1, LINC01150, MIR223HG, AC010980.2, MIR223HG, AC027031.2, AC010980.2, and AC012645.3, have not been reported yet, which could identify new indicators for subsequent study.

To improve risk prediction accuracy and efficacy, we performed an improved Lasso penalized modeling method [20]. Factors were incorporated into a Cox regression according to occurrence frequency rank in the iteration process rather than intersection, because occurrence frequency indicates the influence of this factor on the signature. In addition, we improved the modeling process as follows: we identified the maximum AUC value as an ideal model and compared it with other clinical setting. Besides, we calculated every AIC value to obtain an ideal cut-off point rather than differentiating the risk group merely by the median value. Based on the cut-off point, we differentiated patients into different risk groups, and then used univariate and multivariate analyses and Kaplan–Meier log-rank test of the clinicopathological characteristics, which indicated that the constructed modeling algorithm worked well.

Current studies have shown the functional relevance of lncRNAs to tumor-infiltrating immune cell and immunity regulation in NSCLC [21]. Consequently, irlncRNAs could act as biomarkers for survival prognosis and be promising therapeutic targets for LUAD [22]. Further, other research have suggested that immune cell infiltration is closely correlated with prognosis. For example, Wang et al. [23] established a LUAD prognostic nomogram comprising immune-infiltrating Treg-related genes. In addition, DHX37 influences the prognosis of LUAD through immune cell infiltration [24]. Moreover, Fan et al. [25] demonstrated that multiple immune cell subtypes infiltration was closely related to LUAD prognosis.

To study the correlation between infiltration of immune cells and risk score, we performed seven common accepted software to evaluate the immune cell infiltration. By integrating the analysis, the results suggested that risk score were significantly negatively correlated with several immune cell subtypes infiltration. Meanwhile, increasing research has confirmed the close correlation between TME and tumorigenesis and progression [26]. To explore the stromal cell and immune cell content around tumors in patients with LUAD, we calculated the patients’ StromalScore, ImmuneScore, and ESTIMATEScore, which indicated that the risk score and stromal cells and immune cells content were significantly negatively correlated.

GSEA confirmed robust correlation between the prognosis model and several immune-related pathways. The low-risk patients may be more active in the following immune activities: the IgA production in intestinal immune network and the T and B cell receptor signaling pathway. We speculated that LUAD patients with high risks may promote the progression of LUAD through the activation of the following pathways, leading to poor prognosis: cell cycle, P53 signaling, DNA replication, adhesion junctions, actin cytoskeleton regulation, cancer pathways, and TGF signaling. P53 [27], TGF-β [28] and RHPN2 [29] participate in LUAD occurrence and development.

Platinum-containing dual drug chemotherapy is the cornerstone of advanced LUAD treatment [30]. Unfortunately, drug resistance reduces chemotherapy chemosensitivity dramatically, medication for patients with LUAD become tricky [31]. Therefore, screening high-sensitivity anti-tumor drugs is crucial for LUAD treatment. Here, we separately assessed the sensitivity to common chemotherapy drugs and targeted therapy drugs in different risk groups to guide clinical medication. In recent years, cancer immunotherapy has revolutionized LUAD treatment [32]. Immunotherapies (PD-1, PD-L1, CTLA-4 inhibitors) have shown long-term remission in NSCLC [33]. To better estimate the efficacy of checkpoint blockade therapy on our model, we evaluated CTLA-4, HAVCR2, PD-L1, and PD-1 expression levels in the model. We found that the signature acted as a promising predictor for the selection of anti-tumor drugs.

There are several limitations to our study. First, the bias of the profile analyzed cannot be ignored, considering the data were acquired from public resources. Second, owing to lacking detailed clinical information and insufficient newly identified lncRNAs in the Gene Expression Omnibus (GEO) database, we can hardly find an ideal GEO dataset that included both expression of the 12 DEirlncRNAs and reliable prognostic information. Third, there are currently no immunotherapy drugs for lung cancer in the pRRophetic package, so the sensitivity of patients with lung cancer to immunotherapy drugs requires further exploration. Finally, external validation involving the gene expression of patients with LUAD, such as quantitative real-time PCR and microarrays, are required to confirm our findings.

In conclusion, we discovered a prognostic signature based on DEirlncRNA pairs that accurately predicts the survival outcomes of patients with LUAD without requiring precise expression levels. A high prognostic signature risk score correlates with poor prognosis, whereas a low score correlates with better prognosis. These data provide valuable insights for future investigations into potential individualized treatments for patients with LUAD in different risk groups.
